# Reversible Modulation of Myofibroblast Differentiation in Adipose-Derived Mesenchymal Stem Cells

**DOI:** 10.1371/journal.pone.0086865

**Published:** 2014-01-23

**Authors:** Vivek D. Desai, Henry C. Hsia, Jean E. Schwarzbauer

**Affiliations:** 1 Department of Molecular Biology, Princeton University, Princeton, New Jersey, United States of America; 2 Robert Wood Johnson Medical School, Rutgers, The State University of New Jersey, New Brunswick, New Jersey, United States of America; University of California, San Diego, United States of America

## Abstract

Unregulated activity of myofibroblasts, highly contractile cells that deposit abundant extracellular matrix (ECM), leads to fibrosis. To study the modulation of myofibroblast activity, we used human adipose-derived mesenchymal stem cells (ADSCs), which have much potential in regenerative medicine. We found that ADSCs treated with TGF-β developed a myofibroblastic phenotype with increases in α-smooth muscle actin (α-SMA), a myofibroblast marker, and ECM proteins type I collagen and fibronectin. In contrast, treatment with bFGF had the opposite effect. bFGF-differentiated ADSCs showed marked down-regulation of α-SMA expression, collagen I, and fibronectin, and loss of focal adhesions and stress fibers. Functionally, bFGF-differentiated ADSCs were significantly more migratory, which correlated with up-regulation of tenascin-C, an anti-adhesive ECM protein, and vimentin, a pro-migratory cytoskeletal protein. On the other hand, TGF-β-differentiated ADSCs were significantly more contractile than bFGF-differentiated cells. Interestingly, cells completely reversed their morphologies, marker expression, signaling pathways, and contractility versus migratory profiles when switched from culture with one growth factor to the other, demonstrating that the myofibroblast differentiation process is not terminal. Cell differentiation was associated with activation of Smad2 downstream of TGF-β and of ERK/MAP kinase downstream of bFGF. Reversibility of the TGF-β-induced myofibroblastic phenotype depends, in part, on bFGF-induced ERK/MAP kinase signaling. These findings show that ADSC differentiation into myofibroblasts and re-differentiation into fibroblast-like cells can be manipulated with growth factors, which may have implications in the development of novel therapeutic strategies to reduce the risk of fibrosis.

## Introduction

In response to transforming growth factor-β1 (TGF-β), fibroblasts and other mesenchymal cells differentiate into myofibroblasts. These highly contractile cells are characterized by up-regulation of the extracellular matrix (ECM) proteins type I collagen and fibronectin, increased α-smooth muscle actin (α-SMA) expression, and robust stress fibers and focal adhesions [1]. Potential precursors for myofibroblasts include fibroblasts, mesenchymal stem cells, smooth muscle cells, endothelial cells, and fibrocytes [2]. Myofibroblasts are dramatically increased during wound repair [3], [4], [5]. After the contraction phase of repair, their numbers are reduced through apoptosis [6], but other processes may also contribute to the loss of these cells. For example, reversal of the myofibroblastic phenotype has been reported [7], [8]. Regardless of the actual means by which myofibroblasts disappear, failure of this process and the persistence of cells with a myofibroblastic phenotype are associated with the development of fibrosis, marked by excessive ECM deposition and unregulated contraction [1], [9]. Since ECM amount and stiffness are critical for the structural and functional integrity of tissues, excess ECM in fibrosis results in the development and exacerbation of tissue dysfunction [10]. Thus, a deeper understanding of how the myofibroblastic phenotype can be modulated and by what mechanisms might allow for the development of new therapeutic approaches.

TGF-β is considered the major inducer of myofibroblast differentiation [11], whereas, in some cell types, basic fibroblast growth factor (bFGF) has been shown to down-regulate myofibroblastic features such as α-SMA expression and contraction, thereby enhancing a more fibroblast-like phenotype [7], [8], [12]. Here we show the effects of these two growth factors on differentiation by human adipose-derived mesenchymal stem cells (ADSCs) and compare the features of TGF-β-differentiated myofibroblast-like cells with bFGF-differentiated fibroblast-like cells, all derived from ADSCs. This cell type has gained much attention for use in regenerative medicine because of its relative ease of isolation compared to other stem cells and enormous therapeutic potential [13], [14]. For example, in soft tissue repair applications, supplementation of autologous fat grafts with ADSCs reportedly improves graft viability and retention [15], [16].

Like other mesenchymal cells, ADSCs respond to TGF-β by up-regulating α-SMA expression and other features associated with myofibroblasts [17], [18], [19]. TGF-β also has an inductive role in chondrogenic and osteogenic lineage differentiation by ADSCs [20], [21]. The effects of bFGF on ADSCs are less well-established. bFGF appears to modulate chondrogenic differentiation [22], [23] and it reportedly regulates self-renewal of human ADSCs [24]. However, the role of bFGF in ADSC differentiation related to fibrogenic processes has not been described.

Our data demonstrate that the fibrogenic potential of ADSCs, including changes in the ECM, the cytoskeleton, and cell signaling, can be modulated, and a constellation of features associated with a myofibroblastic phenotype are enhanced by TGF-β and suppressed by bFGF. Furthermore, our results show a requirement for bFGF and activation of extracellular signal-regulated kinase/mitogen-activated protein (ERK/MAP) kinase in the re-differentiation of myofibroblasts to a fibroblastic phenotype. Our findings have implications in regenerative medicine as selective growth factor treatment may be a potent way to manipulate the ADSC phenotype in soft tissue repair processes.

## Results

### Changes in Cytoskeletal Features and Cell Signaling in Response to TGF-β and bFGF

**Figure 1:**
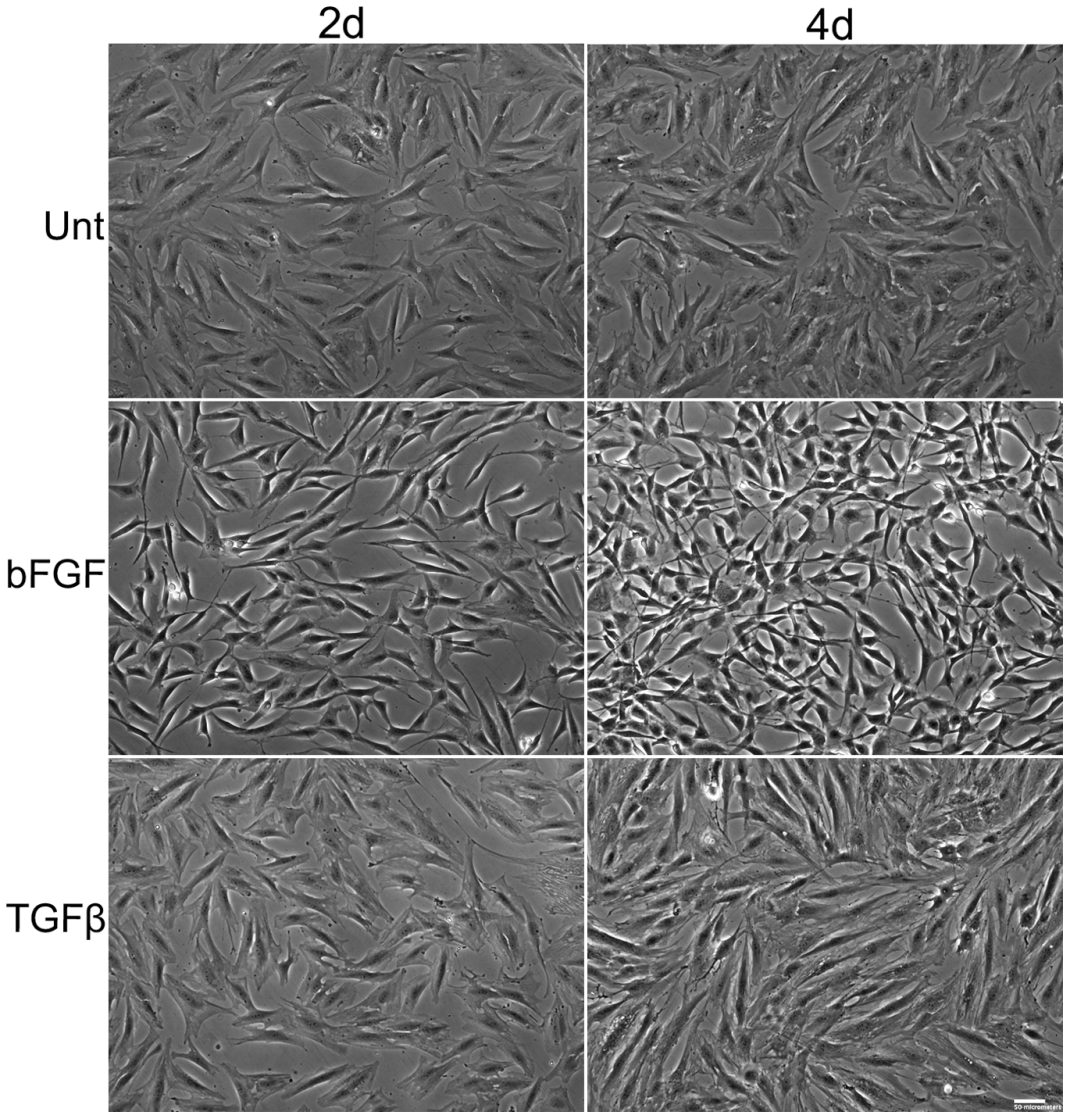
Differences in cell morphology with TGF-β and bFGF. ADSCs in SSFM were treated with 10/mL bFGF plus 5 µg/ml heparin (bFGF), 1 ng/mL TGF-β1 (TGFβ), or no additions (Unt). Phase contrast images were taken at 2 days (2d) and 4 days (4d) of treatment. Images are representative of more than three independent experiments. Scale bar = 50 µm.

Changes in cell shape accompany myofibroblast differentiation [25] so we compared morphologies of ADSCs treated with TGF-β or bFGF. All treatments were carried out in serum-free medium. With bFGF treatment, ADSCs became spindle-shaped and exhibited a dendritic morphology compared to TGF-β-treated cells, which were more spread (Figure 1). Cell populations showed uniform morphologies by 4 days so this regimen of growth factor treatment was used unless otherwise stated.

**Figure 2:**
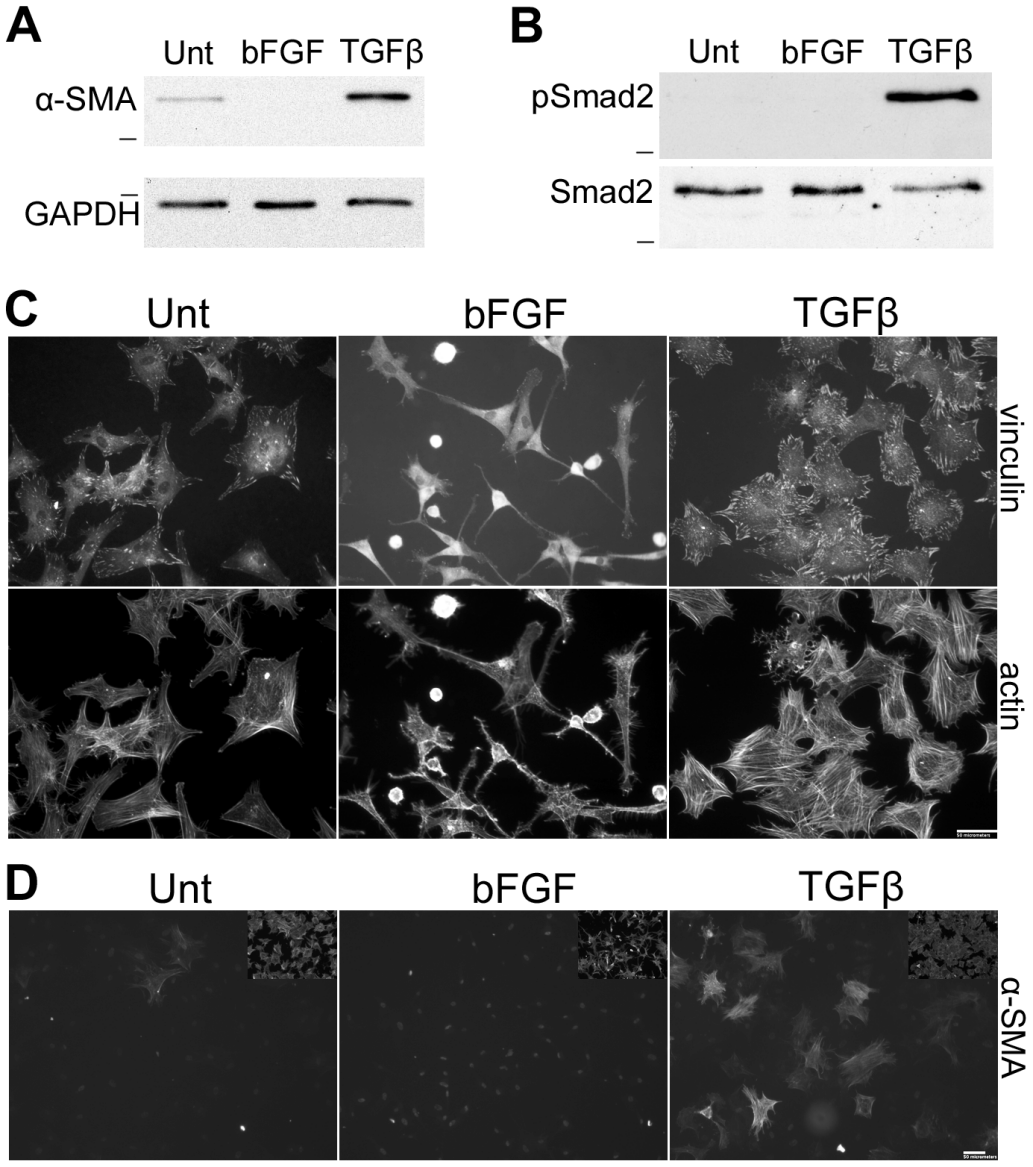
Cytoskeletal responses to TGF-β and bFGF. Cell lysates prepared from ADSCs treated with TGFβ, bFGF or untreated (Unt) were subjected to SDS-PAGE and immunoblotted. (A) 4-day lysates were probed with anti-α-SMA monoclonal antibody or anti-GAPDH antibody as a loading control. Blot is representative of three independent experiments. (B) Lysates prepared at 1 hour of treatment were immunoblotted with anti-phospho-Smad2 (pSmad2) and with total Smad antibody. Blot is representative of three independent experiments. Dash represents 37 kD (A) or 50 kD (B). (C, D) After 4 days of treatment, cells were replated onto collagen-coated glass coverslips for two hours before fixation, permeabilization, and staining with anti-vinculin antibody (C, top), rhodamine-phalloidin (C, bottom), or anti-α-SMA monoclonal antibody (D). Insets in (D) show rhodamine-phalloidin staining of the same fields. All images are representative of three independent experiments. Scale bars = 50 µm.

Myofibroblast differentiation is routinely gauged by α-SMA expression. Analysis of whole cell lysates prepared from treated ADSCs showed that TGF-β enhanced α-SMA protein levels by 6-fold compared to ADSCs left untreated for the same time period (6.1+/−1.9, n = 3, p<0.05) (Figure 2A). In contrast, bFGF treatment suppressed α-SMA expression compared to untreated ADSCs. Smad signaling is the canonical pathway responsible for TGF-β-induced up-regulation of α-SMA expression [11]. To examine Smad2 activity, whole cell lysates of treated or untreated ADSCs were harvested after 1 hour of treatment with growth factors. Immunoblots with anti-phospho-Smad2 (Ser465/467) antibody detected phospho-Smad2 in ADSCs treated with TGF-β but not in those treated with bFGF or in untreated control cells (Figure 2B). This difference in Smad2 activation between TGF-β and bFGF treatments most likely explains the downstream changes in α-SMA expression.

Myofibroblasts are highly contractile cells with prominent stress fibers and focal adhesions [1], [2]. To analyze these cytoskeletal features, differentiated ADSCs were fixed and stained using a monoclonal antibody against the focal adhesion protein vinculin and fluorescent phalloidin to visualize the actin cytoskeleton. The TGF-β-differentiated ADSCs showed much more robust focal adhesions and more pronounced actin stress fibers compared to bFGF-differentiated or untreated ADSCs (Figure 2C). In contrast, bFGF-differentiated ADSCs were less spread and had almost no focal adhesions even when compared with the control untreated ADSCs. α-SMA-positive stress fibers were detected in a small percentage of untreated ADSCs; the percentage of positive cells per field increased 4.5-fold with TGF-β-treatment (5.6±1.2 vs 25.3±5.3, n = 3, p<0.01), as determined by immunofluorescence with anti-α-SMA monoclonal antibody (Figure 2D). bFGF treatment eliminated α-SMA-positive stress fibers.

The α-SMA results combined with the uniformity of cell morphologies of differentiated cells indicate that TGF-β induces ADSCs to differentiate into myofibroblast-like cells, whereas bFGF induces differentiation into fibroblast-like cells. The following experiments primarily characterize and compare these two phenotypes.

### Opposing Effects of TGF-β and bFGF on the Expression of ECM Proteins

**Figure 3:**
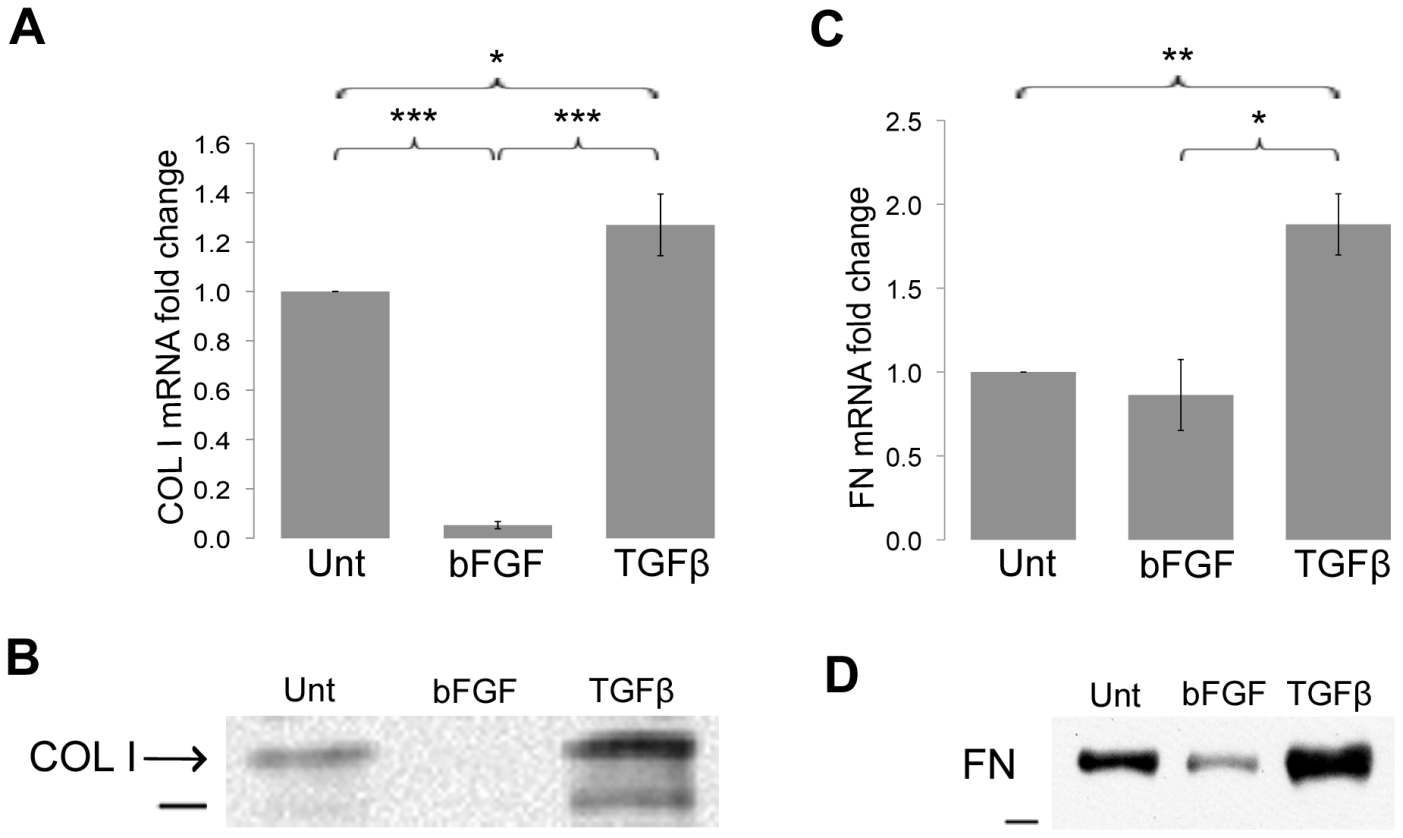
Effects of growth factors on ECM expression. Total RNA was isolated from untreated (Unt), bFGF-treated, or TGF-β-treated ADSCs after 4 days of treatment and qRT-PCR was performed for type I collagen (A) and fibronectin (C) using ubiquitin C as the normalization control. Graphs represent the average of multiple samples from three independent experiments; error bars indicate SEM. *p<0.05, **p<0.01, and ***p<0.001. (B) Anti-type I procollagen antibody was used to detect collagen in conditioned media separated in an 8% polyacrylamide-SDS gel. Sample loadings were normalized to total RNA amounts isolated from the cells. Blot is representative of three independent experiments. (D) Equivalent amounts of protein in urea lysates were separated in a 5% polyacrylamide-SDS gel, and fibronectin was detected with an anti-fibronectin monoclonal antibody. Anti-GAPDH was used to confirm equal loading (not shown). Blot is representative of two independent experiments. Dash represents 150 kD (B) or 250 kD (D).

Excessive production of type I collagen and fibronectin is considered a hallmark of fibrosis in vivo and these ECM proteins are deposited primarily by myofibroblasts [1]. To correlate our observations in cytoskeletal and cell signaling changes with changes in ECM expression, we first analyzed the impact of TGF-β and bFGF on type I collagen expression via quantitative reverse transcription polymerase chain reaction (qRT-PCR). TGF-β caused an expected increase in collagen I mRNA expression compared to untreated ADSCs (Figure 3A). In contrast, bFGF treatment reduced collagen I mRNA a dramatic 20-fold relative to untreated ADSCs. We also measured extracellular type I collagen protein in the cell culture medium. Medium conditioned for two days was analyzed by immunoblot using an antibody against type I procollagen, which showed that cells treated with TGF-β had >6-fold more collagen I in the medium than did untreated cells (6.3+/−0.99, n = 3, p<0.01) (Figure 3B). No type I collagen was detected in the medium of bFGF-differentiated ADSCs.

Growth factor treatments had similar effects on levels of fibronectin. TGF-β up-regulated fibronectin mRNA compared to untreated cells (1.9-fold) or bFGF-treated cells (2.2-fold) (Figure 3C). Immunoblot analysis of cell lysates showed >3-fold more fibronectin with TGF-β treatment than bFGF treatment (3.4+/−0.7, n = 2, p<0.05) (Figure 3D). Taken collectively, these results show that TGF-β up-regulates, whereas bFGF down-regulates, type I collagen and fibronectin expression in ADSCs.

### A Migratory Phenotype is Induced by bFGF

**Figure 4:**
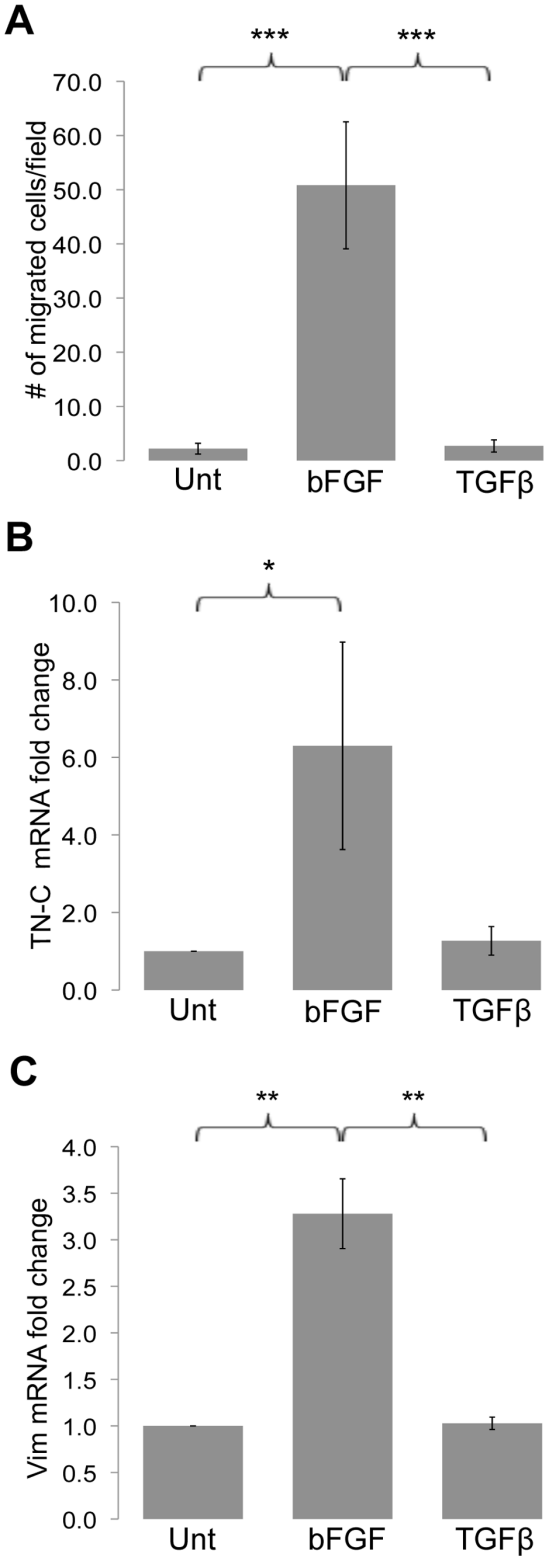
bFGF induces a migratory cell phenotype. (A) Migration of ADSCs either untreated (Unt) or treated with bFGF or TGF-β for four days was measured using a Transwell migration assay. Cells were plated on a type I collagen-coated Transwell filter in SSFM. SSFM containing 2% serum was placed in the bottom chamber and cells were allowed to migrate for 21.5 hours. Cells were fixed and stained with DAPI and counted by fluorescence microscopy. Quantification represents the average number of cells per field +/− SEM from four independent experiments. ***p<0.001. (B, C) Total RNA was isolated from untreated (Unt), bFGF-treated, or TGF-β-treated ADSCs after 4 days of treatment. RNA was used for qRT-PCR with primers for tenascin-C (B) or vimentin (C). Ubiquitin C was used as the normalization control. Graphs represent the average of multiple samples from three independent experiments +/− SEM. *p<0.05, **p<0.01.

α-SMA expression has been shown to restrain fibroblast migration [26]. Migration of growth factor-differentiated ADSCs was compared in a Transwell migration assay. Migration by bFGF-differentiated fibroblast-like cells was ∼23-fold higher than untreated ADSCs and ∼19-fold higher than TGF-β-differentiated myofibroblast-like cells (Figure 4A). Tenascin-C is an anti-adhesive ECM protein that is known to modulate cell migration [27] and to trigger loss of focal adhesions [28]. Analysis by qRT-PCR showed a marked increase in tenascin-C expression by bFGF compared to both untreated cells and myofibroblast-like cells (Figure 4B). A cytoskeletal change was also noted in vimentin, an intermediate filament protein with an important role in cell motility and wound repair [29], [30]. Vimentin mRNA expression was up-regulated about 3-fold in migratory bFGF-treated cells, but not in myofibroblast-like cells (Figure 4C). These results show molecular changes that accompany migratory activity, an important functional difference between fibroblast-like and myofibroblast-like cells.

### Myofibroblast Differentiation of ADSCs is Reversible

**Figure 5:**
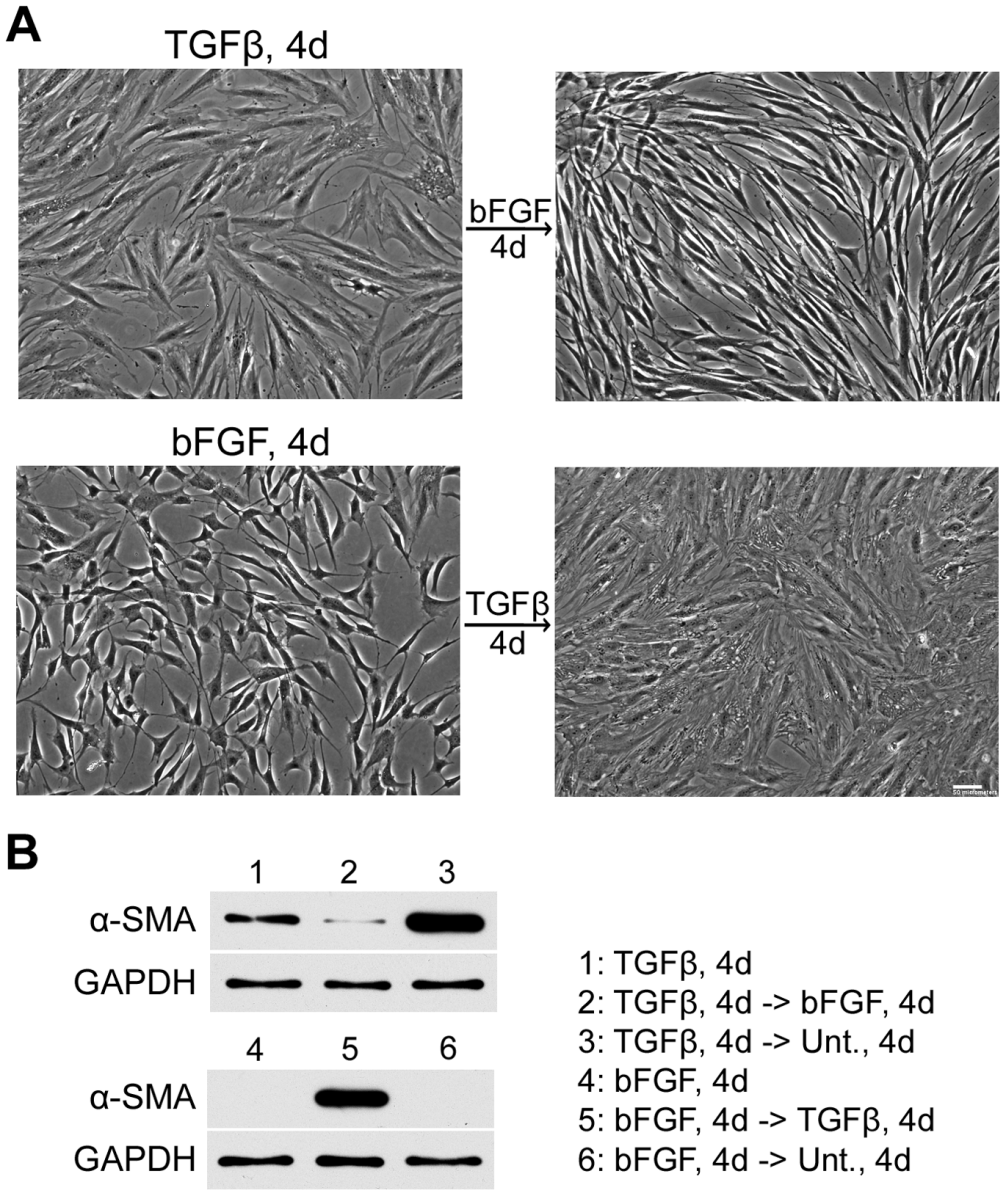
Growth factor-induced myofibroblast differentiation is reversible. (A) Phase images are shown of ADSCs treated with TGF-β (TGF-β, 4d) or with bFGF (bFGF, 4d) for 4 days (left). SSFM containing bFGF was added to TGF-β-differentiated cells and TGF-β was added to bFGF-differentiated cells. Phase images were captured after 4 days (right). Images are representative of more than three independent experiments. Scale bar = 50 µm. (B) Lysates were prepared from cells grown under the six indicated conditions and were used for immunoblots with anti-α-SMA monoclonal antibody. GAPDH was used as the loading control. Blot is representative of three independent experiments. Legend indicates cell treatments in lanes 1–6. Unt., 4d = untreated for 4 days.

Myofibroblasts usually disappear as healing progresses [6], and their unregulated persistence is strongly linked to excessive ECM deposition and contraction, defined as fibrosis [9]. Reversing the myofibroblastic phenotype may thus provide a strategy to develop therapeutic approaches in order to prevent fibrotic complications. Treatment of TGF-β-differentiated myofibroblast-like cells with bFGF caused a complete switch of cell morphological features. Myofibroblast-like cells have a well-spread morphology as expected; however, when switched to the bFGF treatment, the cells assume a more elongated and “dendritic” morphology (Figure 5A). In contrast, when bFGF-differentiated fibroblast-like cells were treated with TGF-β, they assumed a well-spread morphology. In both cases, the switch in cell morphology was noticeable at day 2 of reverse growth factor treatment (data not shown) and was dramatically different at day 4, so this time point was used for further analyses.

The morphological changes were accompanied by changes in α-SMA expression. Treatment of myofibroblast-like cells with bFGF caused a marked suppression of α-SMA expression (Figure 5B, lane 2). Interestingly, α-SMA expression increased in myofibroblast-like cells over 4 days after removal of TGF-β (Figure 5B, lane 3). This could be due to endogenous production of TGF-β by myofibroblast-like cells (see below). Most importantly, these results show that the reduction in α-SMA expression is an effect of bFGF and not solely due to the absence of TGF-β.

Treatment of bFGF-differentiated fibroblast-like cells with TGF-β resulted in increased α-SMA expression (Figure 5B, lane 5). Exogenous TGF-β is needed to induce an increase in α-SMA expression since fibroblast-like cells left untreated for the next 4 days did not increase α-SMA expression (Figure 5B, lane 6). These results show that neither the myofibroblastic nor the fibroblastic phenotype is terminal, as determined here by α-SMA expression. The appropriate growth factor treatments can reverse α-SMA expression levels in both myofibroblast-like cells and fibroblast-like cells.

### bFGF Signaling is Required for Reversal of the Myofibroblastic Phenotype

**Figure 6:**
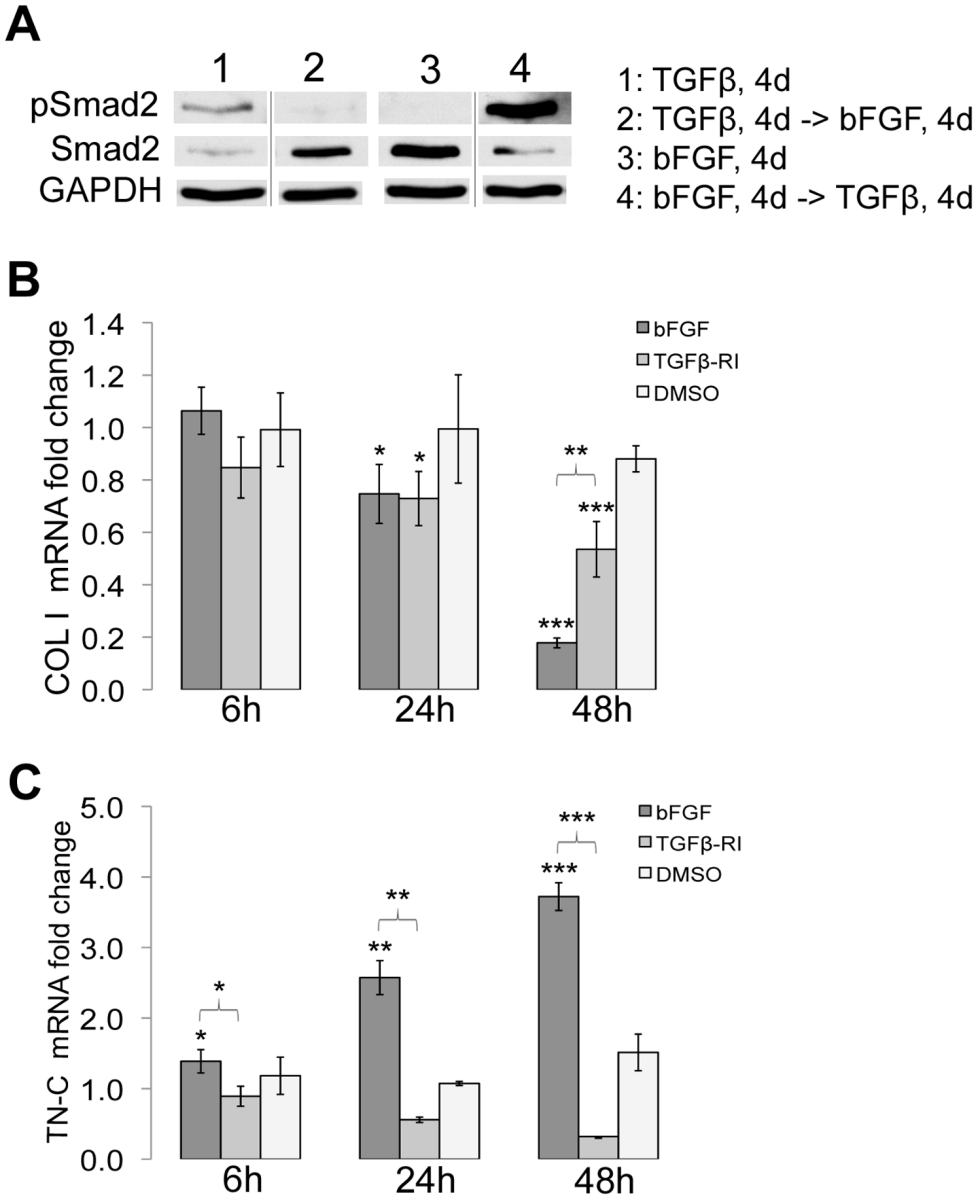
Inhibition of TGF-β-receptor signaling is not sufficient to reverse ECM protein expression. (A) Lysates were harvested for cells grown as indicated in the legend for lanes 1–4, and immunoblotted with anti-phospho Smad2 or anti-total Smad antibody. GAPDH was used as the loading control. Blot is representative of three independent experiments. (B, C) bFGF, TGF-β receptor inhibitor (TGFβ-RI), or DMSO was added to TGF-β-differentiated ADSCs on day 4. Total RNA was isolated at 6, 24, and 48 hours of treatment and qRT-PCR was performed for type I collagen (B) and tenascin-C (C) expression relative to expression in TGF-β-differentiated ADSCs on day 4, using ubiquitin C as the normalization control. Graphs represent the average of multiple samples from three independent experiments +/− SEM. *p<0.05, **p<0.01, and ***p<0.001. The p-values are for comparisons to expression in TGF-β-differentiated ADSCs, unless otherwise indicated.

To determine whether changes in phospho-Smad correspond with α-SMA levels, we examined levels of phospho-Smad2 (Ser465/467) during re-differentiation. Even at 4 days of TGF-β treatment, differentiated cells had detectable phospho-Smad2 (Figure 6A, lane 1). Indeed, phospho-Smad2 was lost in myofibroblast-like cells when treated with bFGF (Figure 6A, lane 2), whereas fibroblast-like cells (Figure 6A, lane 3) treated with TGF-β had increased phospho-Smad2 (Figure 6A, lane 4). The higher levels of phospho-Smad2 in lane 4 than in lane 1 suggest that components of the Smad signaling pathway differ between bFGF-differentiated cells and untreated ADSCs. Interestingly, total Smad2 levels are inversely proportional to the phospho-Smad2 levels, which may be due to proteasome-mediated turnover of phospho-Smad2 [31].

The observed increase in α-SMA levels after removal of TGF-β (see Figure 5B) could result from slow turnover of α-SMA mRNA and protein or from continued cell stimulation by endogenous production of TGF-β. We therefore determined if inhibiting TGF-β receptor signaling was sufficient to drive re-differentiation. We monitored changes in mRNA levels for type I collagen and tenascin-C. TGF-β was removed after 4 days and TGF-β-differentiated myofibroblast-like cells were then treated with bFGF, TGF-β-receptor I kinase inhibitor II, or DMSO, the drug solvent, for 6, 24, or 48 hours. RNA was isolated, and qRT-PCR was performed to measure changes in gene expression relative to that of TGF-β-differentiated ADSCs. By 24 hours, there was a significant decrease in type I collagen mRNA in cells treated with bFGF or with TGF-β receptor inhibitor. By 48 hours, type I collagen mRNA was dramatically decreased, more than 5-fold, in bFGF-treated cells whereas the decrease was less than 2-fold in cells treated with the TGF-β receptor inhibitor (Figure 6B). However, this reduction by the TGF-β receptor inhibitor was significantly more than in DMSO-treated cells, which indicates that in the absence of exogenous TGF-β, endogenous TGF-β has a role in maintaining type I collagen expression. Importantly, removal of exogenous TGF-β or inhibition of endogenous TGF-β receptor signaling is not sufficient to reverse the myofibroblastic phenotype and addition of bFGF is needed.

Analyses of tenascin-C expression led to a similar conclusion. bFGF treatment induced a significant increase in tenascin-C mRNA as early as 6 hours (Figure 6C), while by 48 hours, tenascin-C mRNA was up-regulated almost 4-fold. No increase in tenascin-C expression was observed with the TGF-β receptor inhibitor. Collectively, these results show that loss of TGF-β receptor signaling is not sufficient to reverse ECM protein expression in myofibroblast-like cells. Apparently, bFGF has an active role in upregulating tenascin-C expression and suppressing the myofibroblastic phenotype.

### bFGF Reverses Tenascin-C up-regulation through ERK/MAP Kinase

**Figure 7:**
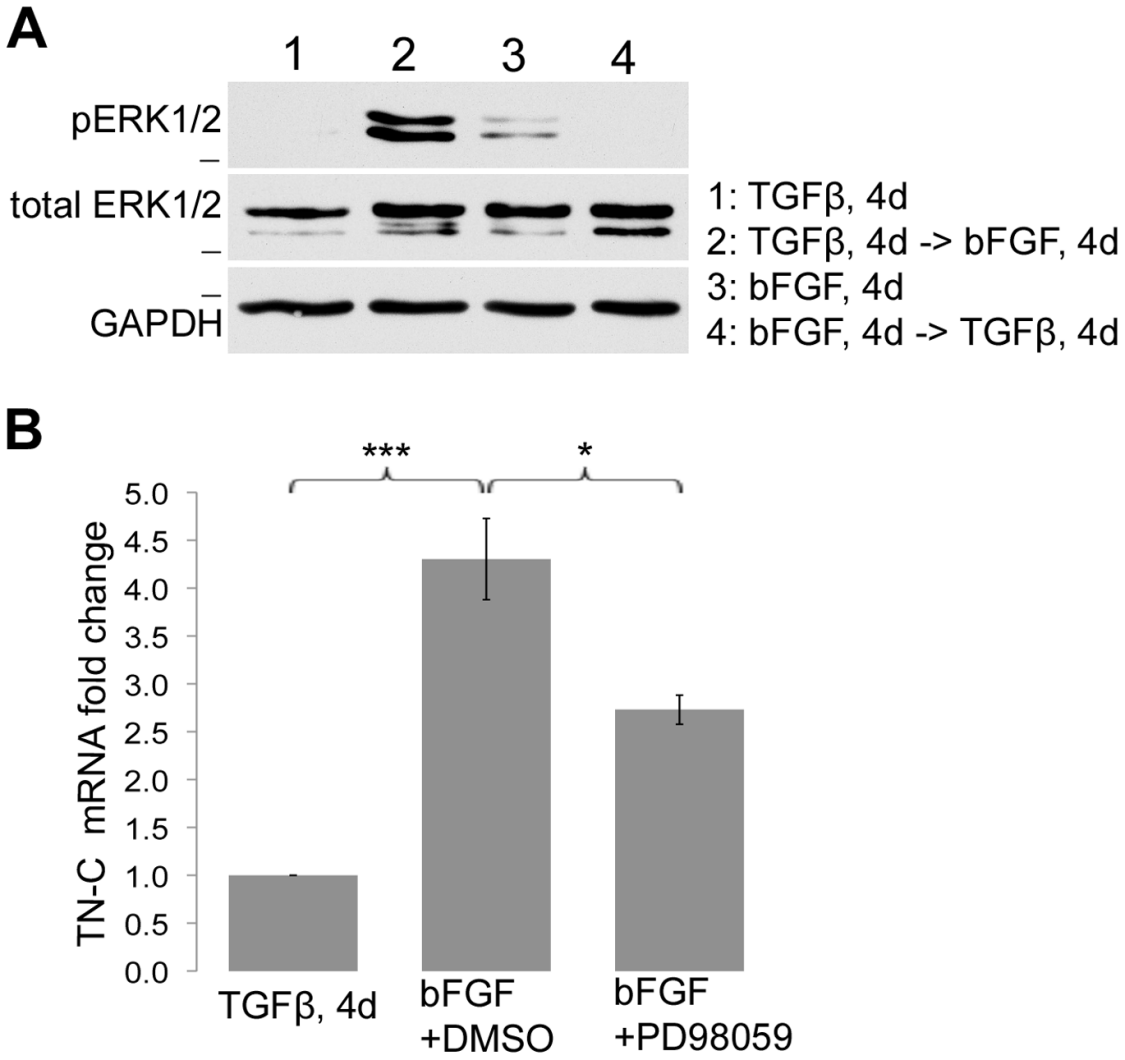
bFGF and ERK/MAP kinase induce tenascin-C expression. (A) Lysates were harvested after growth factor treatment as indicated in the legend for lanes 1–4. Immunoblots were probed with phospho-specific anti-ERK1/2 monoclonal antibody or anti-total ERK antibody. GAPDH was used as the loading control. Blot is representative of three independent experiments. Dash represents 37 kD. (B) bFGF, along with 20 µM PD98059 or DMSO, was added to TGF-β-differentiated ADSCs at day 4. Total RNA was isolated after 48 hours of treatment and qRT-PCR was performed for tenascin-C using ubiquitin C as the normalization control. Graphs represent the average of multiple samples from three independent experiments +/− SEM. *p<0.05, ***p<0.001.

The ERK/MAP kinase pathway is activated by bFGF in many cell types [32]. We examined the levels of active ERK1/2 in growth factor-differentiated ADSCs by immunoblotting whole cell lysates with a phospho-specific anti-ERK1/2 antibody. Phospho-ERK1/2 was present in bFGF-treated fibroblast-like cells that were differentiated from ADSCs or from TGF-β-differentiated myofibroblast-like cells after 4 days of treatment with bFGF (Figure 7A, lanes 2 and 3). We observed higher phospho-ERK levels when cells had previously been treated with TGF-β compared to untreated ADSCs, which is most likely due to molecular differences between the untreated and growth factor-treated cells. In contrast, no phospho-ERK was detected in cells differentiated with TGF-β (Figure 7A, lanes 1 and 4). Total cellular ERK was not different between the different conditions. This shows that bFGF treatment activates the ERK/MAP kinase pathway in ADSCs and also in myofibroblast-like cells obtained from ADSCs.

To investigate whether bFGF-induced reversal of the myofibroblastic phenotype is dependent on ERK/MAP kinase activity, we used PD98059, a pharmacological inhibitor of this pathway [33]. Cells differentiated with TGF-β were subsequently treated with bFGF in the presence or absence of PD98059, and qRT-PCR was used to measure expression of tenascin-C after 48 hours of treatment. bFGF significantly increased tenascin-C mRNA over 4-fold (Figure 7B). Inhibition of ERK/MAP kinase activation suppressed the effects of bFGF and dampened the increase in tenascin-C mRNA by 37%. We did not observe an effect of PD98059 on the bFGF-induced reduction in type I collagen mRNA (data not shown). Thus, although activation of ERK/MAP kinase may not be essential for reversal of all myofibroblastic features, it is responsible, in part, for the bFGF-induced up-regulation of tenascin-C as myofibroblast-like cells re-differentiate.

### Myofibroblast Contractility and Fibroblast Migration are Reversible Phenotypes

**Figure 8:**
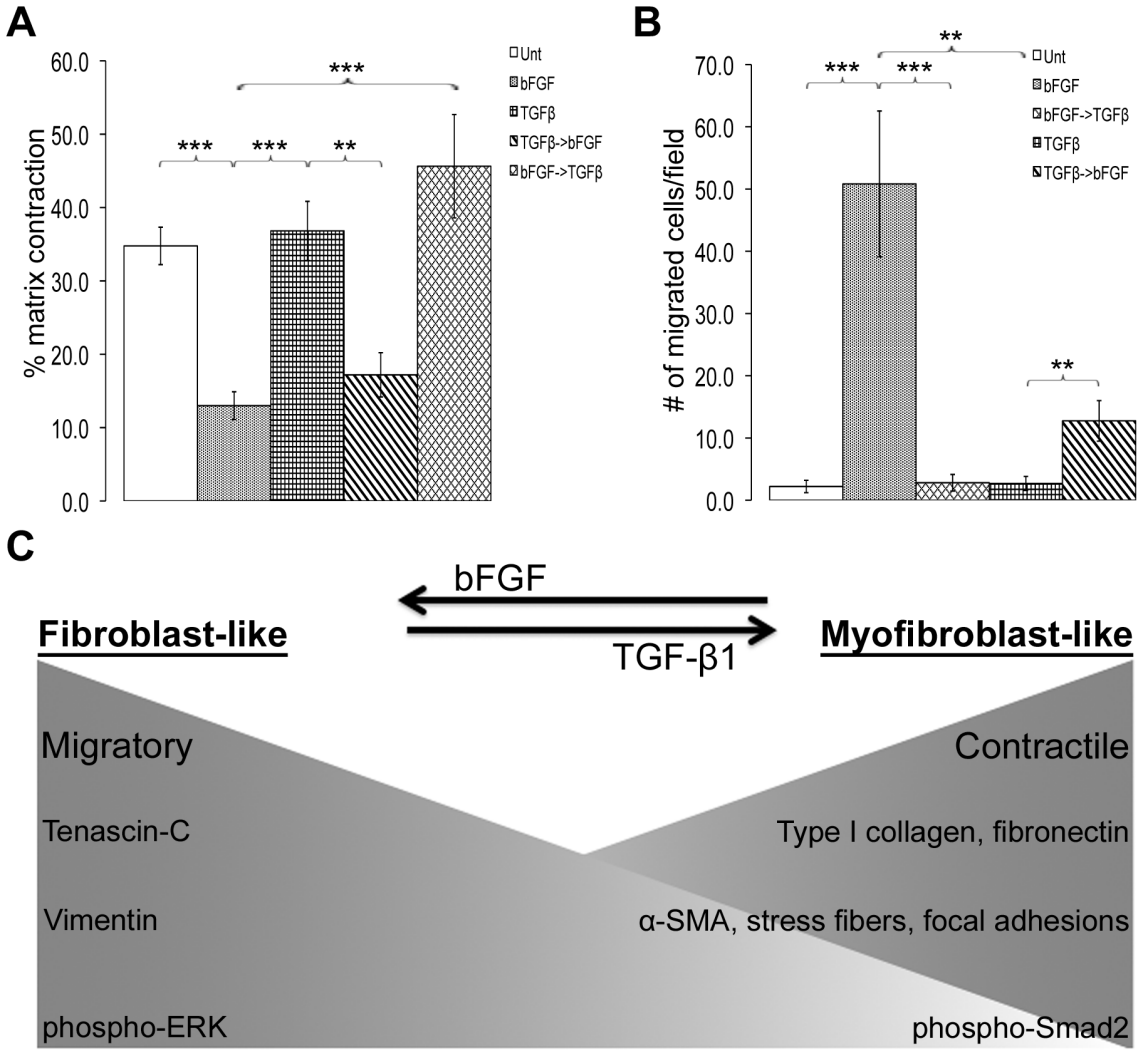
Reversibility of contractile and migratory phenotypes. (A) The % contraction of fibrin-fibronectin matrices by differentiated and re-differentiated cells was averaged for 6–14 total reactions (+/− SEM) from three independent experiments. **p<0.01, ***p<0.001 (B) A Transwell migration assay was performed with re-differentiated cells. Unt, bFGF, and TGFβ data are from Figure 4A. Quantification represents the average number of cells per field +/− SEM from three to four independent experiments. **p<0.01, ***p<0.001. (C) Comparison of myofibroblast and fibroblast features. TGF-β-differentiated ADSCs are myofibroblastic, contractile, and produce increased type I collagen, fibronectin, α-SMA, and phospho-Smad2. Fibroblast-like cells lack myofibroblastic features, are more migratory, and have increased levels of tenascin-C, vimentin, and phospho-ERK1/2. These fibroblastic and myofibroblastic phenotypes are not terminal and are reversed by switching the respective growth factor treatments.

Myofibroblasts assist in closure of the wound and remodeling of the matrix through their increased contractility [1], [2]. Using a well-established fibrin-fibronectin matrix contraction assay [34], we examined whether the morphological and biochemical differences observed between TGF-β- and bFGF-differentiated ADSCs could be correlated with differences in cell contractility. Myofibroblast-like cells, fibroblast-like cells, or untreated cells were added to the matrix components, the matrix was allowed to polymerize and then detached to initiate contraction. bFGF-differentiated fibroblast-like cells had significantly reduced capacity to contract the matrix compared to TGF-β-differentiated myofibroblast-like cells or to untreated cells (Figure 8A). mRNA levels for integrin α5, a major fibronectin receptor, were not different between bFGF- and TGF-β-differentiated cells and both cell types adhere to a fibronectin-coated surface (data not shown) indicating that the differences in matrix contraction are not due to differences in cell-fibronectin interactions.

In re-differentiation studies, bFGF treatment of TGF-β-induced myofibroblast-like cells caused a reduction in their ability to contract the matrix down to a level similar to bFGF-differentiated fibroblast-like cells (Figure 8A). These results show that TGFβ-induced myofibroblast contractility is reversible by treatment with bFGF. Furthermore, TGF-β treatment of bFGF-differentiated fibroblast-like cells caused an enhancement in their ability to contract the matrix to a level similar to myofibroblast-like cells (Figure 8A). This shows that fibroblast-like cells retain the potential to differentiate into contractile myofibroblast-like cells. Identical relative differences in contraction were obtained after 10 min (Figure 8A) and 2 hr (data not shown) of contraction time. Thus, along with the reversibility of the myofibroblast morphology, marker expression, and ECM protein production, the ability to contract a fibrin-fibronectin matrix is also reversed when cells are exposed to bFGF.

Stable cell-ECM contacts, while necessary for contraction, can inhibit cell migration [35], so we postulated that the cell migratory behavior of differentiated ADSCs inversely correlates with cell contractility. Using the Transwell migration assay, we observed an ∼5-fold increase in migration of TGF-β-differentiated myofibroblast-like cells when treated with bFGF (Figure 8B). On the other hand, bFGF-differentiated fibroblast-like cells, when treated with TGF-β, showed a dramatic ∼18-fold decrease in migratory behavior. These results show that when myofibroblasts lose their myofibroblastic features, they become more migratory, and the opposite is true when fibroblast-like cells are induced to differentiate into myofibroblasts.

## Discussion

Understanding the regulation of myofibroblast differentiation has important implications for promoting tissue repair and regeneration and reducing fibrosis. We demonstrate here that ADSCs can be induced to differentiate into either myofibroblast-like or fibroblast-like cells using growth factors present in the wound microenvironment. TGF-β treatment enhanced whereas bFGF treatment decreased a constellation of biochemical features and biological functions associated with the myofibroblastic phenotype (Figure 8C). Of most significance was our finding that the myofibroblastic phenotype is plastic and malleable in that myofibroblasts can be re-differentiated into fibroblast-like cells by bFGF, but not by lack of TGF-β or by inhibition of endogenous TGF-β receptor signaling. We also found that bFGF activation of ERK/MAP kinase contributes to re-differentiation by enhancing the expression of tenascin-C, an anti-adhesive and pro-migratory ECM protein.

We characterized ADSC differentiation by TGF-β or bFGF using a collection of features associated with a myofibroblastic phenotype, including morphology, cytoskeletal features, signaling, ECM production, cell migration, and matrix contraction. Contrary to TGF-β, the effects of bFGF on myofibroblasts remain poorly defined. Studies have shown that bFGF can suppress α-SMA expression in some cell types, including epithelial cells [12] and fibroblasts [7]. bFGF is also known to inhibit wound contraction in animal models [36]. However, matrix contraction ability does not necessarily correlate with α-SMA expression in all cell types; for example, in human lens epithelial cells, knockdown of α-SMA expression did not reduce TGF-β-dependent cell contractility [37]. Our results show consistent changes in biochemical and functional properties of ADSCs during both initial differentiation and subsequent re-differentiation. Our findings are also in accord with the in vivo findings that ADSCs pretreated with bFGF reduced liver fibrosis when transplanted in mice [38].

Even though myofibroblasts are crucial for proper wound repair, their timely disappearance [6] is necessary, otherwise fibrotic complications can result [9]. We show that myofibroblastic and fibroblastic phenotypes obtained from ADSCs are not terminal and can be modulated reversibly and the contrasting interplay observed between bFGF and TGF-β in this modulation may have relevance to in vivo tissue repair. Studies have shown that in other cell types α-SMA expression can be reversed by changing cell density [39], [40], by manipulating tensional forces [41], [42], [43], by changing stiffness [44], or by growth factors [7], [8]. Here, bFGF completely reversed the TGF-β-differentiated myofibroblastic phenotype in ADSCs. Re-differentiation of myofibroblast-like cells was induced by bFGF but not by the lack of TGF-β or by blocking TGF-β receptor signaling.

Our results show that bFGF plays an active role in this process through ERK/MAP kinase to up-regulate pro-migratory proteins tenascin-C and vimentin (unpublished data). Thus, we have established a novel connection between bFGF-induced ERK/MAP kinase activation and tenascin-C expression. Blockade of ERK/MAP kinase activation did not completely prevent tenascin-C expression and did not affect the down-regulation of type I collagen mRNA indicating that other pathways are also involved in re-differentiation. The ERK/MAP kinase pathway may affect translocation of Smad into the nucleus as shown in valvular interstitial cells [45]. It is also possible that bFGF affects actin dynamics to down-regulate α-SMA expression and reduce Rho-mediated contractility, which has been shown to stimulate serum response factor-dependent expression of α-SMA [46]. Signaling pathways downstream of specific ECM proteins may also cooperate with growth factor pathways to control differentiation [47]. These possibilities require further investigation.

Mesenchymal stem cells have potential applications in regenerative medicine and tissue engineering [14]. Recent clinical observations have described therapeutic benefits to patients treated with ADSCs or direct autotransplants of lipoaspirated tissue for fibrotic and wound healing complications arising from a range of diseases including radiotherapy-related skin fibrosis [48], tracheomediastinal fistula [49], and complications of Crohn’s disease [50]. In vitro and animal studies suggest the potential of ADSCs to treat diseases ranging from chronic wounds [51] to post-myocardial infarction complications [52]. Our study gives important insights into the possible behavior of mesenchymal stem cells during wound repair and to their potential utility in regenerative medicine. Our findings suggest that therapeutic strategies can be developed for fibrosis and poor wound healing that more fully exploit the plasticity of ADSCs by driving their myofibroblastic features in a particular direction.

## Materials and Methods

### Cell Culture and Differentiation

Human ADSCs (Invitrogen, Carlsbad, CA) were maintained per the manufacturer’s instructions in MesenPro RS Basal medium containing MesenPro RS Growth Supplement (Invitrogen) and 2 mM L-glutamine (Invitrogen). Cells were passaged at ∼80% confluency with seeding density of 3–3.3×10^5^ cells per 10 cm dish. Medium was changed every 3–4 days. All experiments were performed with cells within 6 passages, as suggested by the manufacturer. The majority of the experiments were carried out with ADSCs at passage 2 or 3. In the few cases where cells at passage 4, 5, or 6 were used, the results were consistent across different passage numbers. ADSCs within 6 passages maintain the ability to differentiate; chondrogenic differentiation in micromass culture is equivalent up through passage 6 (unpublished data).

The differentiation protocol is similar to one described in [53]. All cell treatments were conducted in supplemented serum-free medium (SSFM) in order to determine the effects of each growth factor on ADSCs without the interference of other components. Tissue-culture dishes were coated with 10 µg/mL solution of bovine collagen (Ultrapure) (Sigma, St. Louis, MO) in 2 mL phosphate buffered saline (PBS) for 40 min at 37°C. 2.5×10^5^ ADSCs were plated on 35 mm collagen-coated dishes in 2 mL of SSFM, composed of DMEM:F12 (Invitrogen), ITS Liquid Media Supplement (final concentrations: 10 µg/mL insulin, 5.5 µg/mL transferrin, 6.7 ng/mL selenium) (Invitrogen), RPMI 1640 Vitamin Mix (Sigma), 1 µg/mL glutathione (Sigma), 2 mM L-glutamine (Invitrogen), non-essential amino acids (Invitrogen), sodium pyruvate (Invitrogen), and antibiotic/antimycotic (Invitrogen) [53]. 60 minutes after the cells were plated, 1 ng/mL TGF-β1 (R&D Systems, Minneapolis, MN) or 10 ng/mL bFGF (Invitrogen) +5 µg/ml heparin (Grade I-A, Porcine Intestinal Mucosa, Sigma) was added. For untreated conditions, no growth factors were added. Media were replaced after two days. Unless otherwise stated, cells were analyzed four days after the initiation of growth factor treatment. For re-differentiation experiments, media containing growth factors were removed on day 4 and SSFM+the other growth factor was added. Media were replaced two days later and cells were analyzed after four days.

The following inhibitors were used in this study: TGF-β receptor I inhibitor II (Calbiochem, Billerica, MA, USA) and PD98059 (Enzo Lifesciences, Farmingdale, NY, USA). 20 µM PD98059 was added along with 10 ng/mL bFGF +5 µg/ml heparin. Pre-treatment with PD98059 for 1 hour or the use of higher concentrations PD98059 did not change the results (data not shown). Activities of the TGF-β-receptor inhibitor and of PD98059 were confirmed with control immunoblots of phospho-Smad2 and phospho-ERK1/2, respectively, after 1 hr of treatment (data not shown). DMSO, the drug solvent, was used as a control in all inhibitor experiments. Cells with DMSO alone had the same levels of changes in gene expression as the cells without DMSO (data not shown), confirming no effect of DMSO on the cells.

### Cell Staining and Microscopy

For cytoskeletal structure staining, cells were plated at 1×10^5^ on 10 µg/mL collagen-coated glass coverslips for 2 hours and then fixed and permeabilized as described previously [54]. Focal adhesions were detected with an anti-vinculin monoclonal antibody (Sigma) diluted 1∶400. α-SMA positive stress fibers were detected with anti-α-SMA monoclonal antibody (Sigma Clone 1A4) at 1∶200. Primary antibodies were detected with fluorescein-conjugated goat anti-mouse antibody (Invitrogen) at 1∶500. Actin filaments were detected with rhodamine-phalloidin (Invitrogen) at 1∶500. Coverslips were mounted in FluoroGuard Antifade Reagent (Bio-Rad, Hercules, CA). Staining was visualized with a Nikon Eclipse Ti microscope; images were captured using an ORCA-R2 camera (Hamamatsu) and analyzed with iVision (Biovision) and Adobe Photoshop Elements software.

α-SMA-positive cells were counted after normalizing the minimum and maximum fluorescence intensities of images taken at the same exposure at the same time. Total cells and α-SMA-positive cells were counted in 4–8 fields per condition at 10× magnification and, in most experiments, an additional 3–5 fields were counted at 20× magnification. The percentage of α-SMA-positive cells was calculated by normalizing the number of α-SMA-positive cells to the number of total cells in the respective field.

Phase contrast images were taken with a Nikon Eclipse TS100 microscope equipped with a SensiCam camera (Cooke) and analyzed with iVision software (Biovision). All images were adjusted identically in Adobe Photoshop Elements software.

### Immunoblotting

For analyzing intracellular proteins, cells were lysed in modified radioimmune precipitation buffer: 50 mM HEPES pH 7.5, 150 mM NaCl, 1.5 mM MgCl_2_, 1% Triton X-100, 1% sodium deoxycholate, 0.1% SDS [55] plus protease inhibitor cocktail tablet (Roche) and phosphatase inhibitors, 1 mM sodium orthovanadate and 10 mM sodium pyrophosphate. Total protein concentration was determined using BCA Protein Assay (Pierce, Rockford, IL).

Whole cell lysates with associated ECM were prepared with urea lysis buffer (8 M urea, 2% SDS, 2% β-mercaptoethanol, 0.16 M Tris-HCl, pH 6.8). Proteins were solubilized by boiling for 5 min. Protein concentration was determined by absorbance at 280 nm. Equivalent amounts of protein in urea lysates were separated in a 5% polyacrylamide-SDS gel. Anti-GAPDH antibody (Cell Signaling, Danvers, MA) was used to confirm equal loading.

Cell conditioned medium was used to analyze type I collagen. Culture medium was changed on day 2 of treatment and then collected on day 4. For SDS-PAGE, samples of conditioned media were normalized to cell number based on quantity of RNA isolated from the respective cell layers (see below).

Samples containing equal amounts of protein were resolved by SDS-PAGE, transferred to nitrocellulose membranes, and detected using the following antibodies and dilutions: α-SMA Clone 1A4 (Sigma) at 1∶5000, fibronectin HFN7.1 culture supernatant at 1∶5000 (Developmental Studies Hybridoma Bank, Iowa City, Iowa), collagen (pro-) type I (aminopropeptide)-concentrate SP1.D8 (Developmental Studies Hybridoma Bank, Iowa City, Iowa) used at 0.2 µg/mL, phospho-Smad2 (Ser465/467) (Cell Signaling) at 1∶1000, Smad2/3 (Cell Signaling) at 1∶1000, anti-MAP kinase activated (diphosphorylated ERK-1&2) (Sigma) at 1∶10,000, p44/42 MAPK (Erk1/2) (Cell Signaling) at 1∶1000, and GAPDH at 1∶2000 (Cell Signaling). Secondary antibodies used were goat-anti-mouse and goat-anti-rabbit IgG (Pierce), both at 1∶10,000 dilution. Chemiluminescence was conducted using ECL reagents (Pierce) per manufacturer’s instructions. Blots were scanned and bands quantified using Quantity One software (Bio-Rad). All blots were exposed for at least three different times, and the exposures that yielded signals within the linear range were used for quantification using the Quantity One software. Quantities for band intensities for α-SMA were normalized to GAPDH and the values for three independent experiments were averaged and SEM was calculated.

### Quantitative RT-PCR Analysis

Total RNA was extracted from cells using Qiagen (Germantown, MD, USA) RNeasy Mini Kit as per the manufacturer’s instructions. cDNA was prepared using Superscript II reverse transcriptase (Invitrogen, Grand Island, NY). qRT-PCR was performed with the cDNA, Brilliant II SYBR QPCR Low Rox Master Mix, and forward and reverse primers using the Mx3000P QPCR System (Stratagene, Agilent Technologies, Clara, CA), following the manufacturer’s instructions. Data were analyzed using QPCR Software (Stratagene). Ubiquitin C was used as the normalization control. Values from at least three independent experiments were averaged and SEM was calculated. All primers were designed using MacVector (MacVector, Inc., Cary, NC, USA) with parameters for qRT-PCR. Sequences of the forward and reverse primers used were as follows:

Fibronectin:

forward primer 5′-TGAAAGACCAGCAGAGGCATAAG-3′

reverse primer 5′-CTCATCTCCAACGGCATAATGG-3′

Type I collagen:

forward primer 5′-GGAAAGAATGGAGATGATGGGG-3′

reverse primer 5′-CCAAACCACTGAAACCTCTGTGTC-3′

Tenascin-C:

forward primer 5′-AAGGAGACATCTGTGGAAGTGGAG-3′

reverse primer 5′-TTTGGTGATCTCTCCCTCATCTTC-3′

Vimentin:

forward primer 5′-TGAAGGAGGAAATGGCTCGTC-3′

reverse primer 5′-GTTTGGAAGAGGCAGAGAAATCC-3′

Ubiquitin C:

forward primer 5′-ATTTGGGTCGCGGTTCTT-3′

reverse primer 5′-TGCCTTGACATTCTCGATGGT-3′

### Transwell Cell Migration Assay

5×10^4^ cells were seeded on the upper chamber of a 24-well Transwell (8 µm pore size) filter (BD Biocoat, Franklin Lakes, NJ) coated with 10 µg/mL type I collagen for 40 min at 37°C. 500 µL SSFM+cells were added to the upper chamber and 500 µL SSFM containing 2% fetal bovine serum was added to the bottom chamber. After 21.5 hours incubation at 37°C, cells were scraped from the top of the filter and cells on the bottom of the filter were stained with DAPI. In initial experiments, cell counts were confirmed by co-staining with fluorescent phalloidin. Staining was visualized with a Nikon Eclipse TE2000-U microscope; images were captured using a Q-Imaging camera (Retiga 1300) and analyzed with iVision software (Biovision). Each independent experiment was performed up to four times. Nuclei were counted in 8–10 fields per Transwell from six to eight total Transwells for each condition at 10× magnification. Quantification represents the average number of cells per field +/− SEM.

### Matrix Contraction Assay

Fibrin-fibronectin matrices were prepared as described previously [34], [56], [57] by mixing the following components: 600 µg/mL fibrinogen (America Diagnostica Inc., Greenwich, CT), 30 µg/mL plasma fibronectin, 15 µg/mL coagulation factor XIIIa (Calbiochem-Novabiochem, San Diego, CA), 150 mM sodium chloride, 50 mM calcium chloride, 10 mM Tris-HCl, pH 7.4, and thrombin 2 U/mL. Cells differentiated or re-differentiated with the respective growth factors for 4 days were trypsinized and washed with PBS. 2×10^5^ cells per ml were added to the matrix components. The matrix was allowed to polymerize at 37°C for 30 min, and then SSFM was added for 10 min at 37°C before the matrix was detached from the well walls to allow contraction. Matrix area was measured 10 min or 2 hr post-detachment. % matrix contraction was calculated as: (area pre-detachment – area post-detachment)/(starting area) × 100. The % contraction of fibrin-fibronectin matrices was averaged for 6–14 total reactions from three independent experiments. Identical results were obtained when growth factors were added to the matrix components along with the cells.

### Statistical Analyses

Student’s t-test was used for all statistical analyses, and *p*-values less than 0.05 were considered significant.
